# Investigating heavy metal concentrations in sea snakes (Elapidae: Hydrophiinae) as an outcome of oil spill exposure

**DOI:** 10.1016/j.heliyon.2024.e35954

**Published:** 2024-08-08

**Authors:** Fadi Yaghmour, Fatin Samara, Yehya El Sayed, Areej Mohammed, Elisa Maio, Susannah Philip, Jane Budd, Johannes Els

**Affiliations:** a Hefaiyah Mountain Conservation Centre (Scientific Research Department), Environment and Protected Areas Authority, Sharjah, United Arab Emirates; b Department of Biology, Chemistry and Environmental Sciences, American University of Sharjah, Sharjah, United Arab Emirates; c Department of Industrial Engineering, Engineering Systems Management Program, American University of Sharjah, Sharjah, P.O. Box 26666, United Arab Emirates; d Breeding Centre of Endangered Arabian Wildlife, Environment and Protected Areas Authority, Sharjah, United Arab Emirates

## Abstract

This study reports the concentration of heavy metals in the tissues of stranded sea snakes that died as a result of exposure to an oil spill on the eastern coast of Sharjah, UAE. Given the limited occurrence of stranded sea snakes observed along Sharjah's eastern coast outside this spill incident, we are using strandings collected from the nearby Arabian Gulf coast of Sharjah to compare the levels of heavy metals in sea snakes affected by the oil spill against their non-oiled counterparts. The sample comprised 14 Arabian Gulf Coral Reef Sea Snakes *(Hydrophis lapemoides*), 6 Yellow-bellied Sea Snakes (*Hydrophis platurus*), and 4 Yellow Sea Snakes (*Hydrophis spiralis*). Overall, our results show significantly higher concentrations of Al, Cd, Pb, Cr, Mn, Fe, Ni, Cu and Zn in sea snakes that were mired in oil.

## Highlights

• We examine levels of heavy metals in the tissues of stranded sea snakes.
• The sample included 24 specimens from 3 species.
• Sea snakes were collected from the eastern and western coasts of Sharjah, UAE.
• The sample consisted of clean sea snakes and sea snakes that were exposed to oil spills.
• Significantly higher concentrations of heavy metals observed in oiled snakes.

## Graphical abstract

**Figure:**
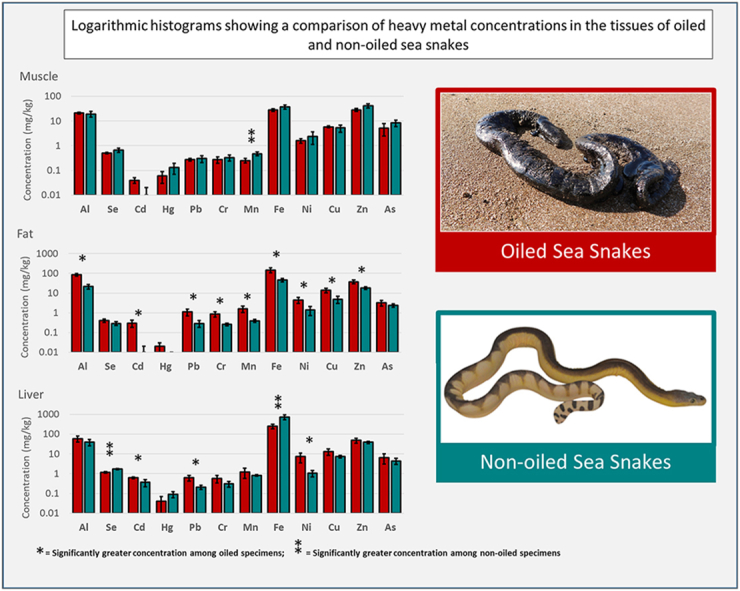

## 1 Introduction

True sea snakes within the subfamily Hydrophiinae are marine viviparous species that spend their entire lives in the oceans. Of the estimated 65 extant species of sea snakes [1], 10 species are recorded within the coastal waters of the Arabian Peninsula of the Gulf of Aden, Arabian Gulf and Gulf of Oman [[2], [3], [4]], while there are no recorded sightings of sea snakes within the Red Sea. Within the Exclusive Economic Zone (EEZ) of the United Arab Emirates (UAE), nine species were recorded in the waters of the Arabian Gulf and Gulf of Oman [[5], [6], [7]], and one species, *Hydrophis viperinus*, is known from a single museum specimen collected in UAE waters, near Sir Bu Na'ir Island, circa 1963 [2]. Environmental contaminants such as heavy metals are considered a great threat to reptile populations worldwide [8]. Heavy metals are defined as any metal or metalloid element with a relatively high density and toxicity even at trace levels [9]. The ability of heavy metals to travel across long distances has made them one of the most ubiquitous pollutants across all marine environments [9]. Heavy metals have the potential to bio-accumulate throughout an animal's life and bio-magnify in higher trophic levels, thereby increasing their potential toxicity to carnivorous taxa [[10], [11], [12], [13]].

Heavy metals are introduced to coastal and marine environments through various sources and activities, including sewage and industrial effluents, bilge discharges, coastal development and oil spills [14]. In the United Arab Emirates (UAE), studies have primarily focused on measuring heavy metal concentrations in sediment and water samples [15,16]. While few investigations have explored the exposure of marine reptiles to various marine pollutants, only one of these specifically examined sea snakes, while none of these studies investigated the presence of heavy metals [[17], [18], [19], [20], [21]]. In fact, there is a global dearth of studies that examine the concentrations of heavy metals in sea snakes. In an investigation conducted by Goiran et al. [22], turtle-headed sea snakes (*Emydocephalus annulatus),* were examined in New Caledonia's inshore bays. The study revealed that sea snakes with darker skin, rich in melanin, effectively bind heavy metals, subsequently expelling them during the shedding process. The study reports higher concentrations of trace elements in both sea snakes dwelling in urbanized waters as well as sea snakes and darker skin. Rezaie-Atagholipour et al. [23], examined the heavy metal exposure of annulated sea snakes (*Hydrophis cyanocinctus*) and their primary prey species (*Periophthalmus waltoni* and *Boleophthalmus dussumieri*) in Iranian waters. Overall, the study found that Cd concentrations were significantly greater in annulated sea snakes compared to their prey species, suggesting that, in the study area, annulated sea snakes undergo biomagnification of Cd via trophic transfer [23]. In a study conducted by Sereshk [24], it was demonstrated that sea snakes from the Arabian Gulf exhibited high concentrations of low molecular-weight PAHs in their skin. The findings suggested that the levels of these compounds in the skin may indicate direct absorption of toxins through the skin. This phenomenon of cutaneous exposure is well-known in terrestrial snakes as a mechanism of exposure, as pointed out by Hopkins [25]. It is reasonable to assume that sea snakes, when directly exposed during oil spills, could potentially experience trans-dermal absorption of contaminants. It has been suggested that metals associated with oil spills are not degraded or metabolized; hence, they bioaccumulate in organisms. Moreover, metal exposure is an important component of oil spills [26].

**Fig. 1:**
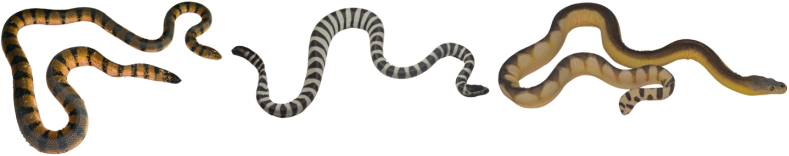
Stranded sea snakes from the coast of Sharjah, United Arab Emirates: *Hydrophis spiralis***[Left]**, *Hydrophis lapemoides***[Middle]** and *Hydrophis platurus***[Right]**. (pictures credit: Johannes Els).

**Fig. 2:**
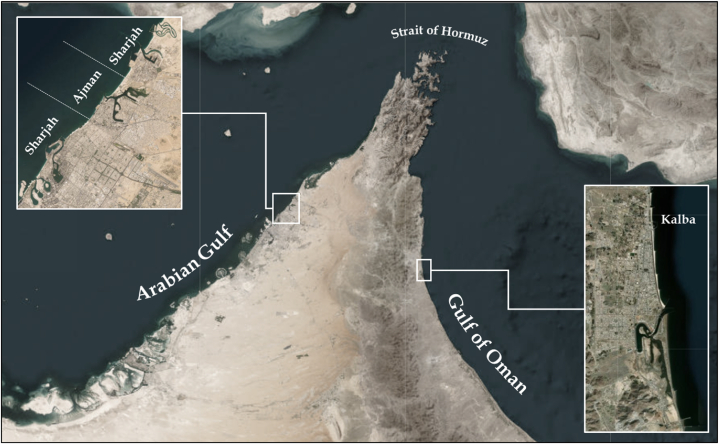
Map of study area along the Arabian Gulf and Gulf of Oman Coast of Sharjah, UAE. Map Images courtesy of Google Earth, earth.google.com/web/.

In this study, we compare the levels of heavy metals from the tissues of oiled sea snakes (described in Ref. [21]) and non-oiled sea snakes from the coasts of Sharjah, UAE. The aim of the present study is to evaluate the qualitative and quantitative aspects of a selection of 12 environmentally relevant heavy metals using tissue samples collected from three species of stranded sea snakes *(Hydrophis lapemoides, Hydrophis platurus,* and *Hydrophis spiralis;* See Fig. 1) from the eastern and western coasts of Sharjah, UAE. In doing so, we assess the concentrations of heavy metals in the tissues of stranded sea snakes from the eastern and western coasts of Sharjah, UAE. Additionally, we aim to investigate whether there are any significant differences in heavy metal accumulation between oiled and non-oiled sea snakes, providing insights into the impact of oil spills on metal exposure in sea snakes (see Fig. 2).

## 2 Methods

### 2.1 Study area

The Emirate of Sharjah encompasses territories located on both the Arabian Gulf and Gulf of Oman shores within the United Arab Emirates (UAE). The Arabian Gulf coast of the Sharjah Emirate includes Sir Bu Na'ir Island and the city of Sharjah. Sir Bu Na'ir Island (25.211815° N; 54.240684° E) is presently designated as a marine protected area, overseen by the Environment & Protected Areas Authority (EPAA), owing to its cultural and ecological significance. The island is renowned for its extensive coral reefs and sandy beaches, which play a crucial role as nesting grounds for hawksbill sea turtles [19]. On the Gulf of Oman coast, the Sharjah Emirate spans from the cities of Kalba, Khor Fakkan, to Dibba Al Hisn. At the southernmost tip of Kalba, near the border with Oman, lies the Alqurm Wa Lhaffaiiah Protected Area (25.013662° N, 56.359,77° E), another protected area overseen by the EPAA. This area is characterized by a tidal inlet featuring channels bordered by gray mangrove trees (*Avicennia marina*).

### 2.2 Sampling

**Fig. 3:**
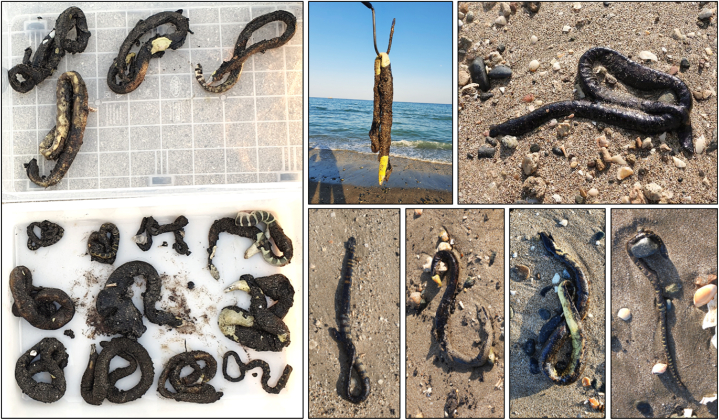
Oiled sea snake strandings observed between 13 and 19/Nov/2021 from the coast of Kalba, on the Gulf of Oman coast of Sharjah, UAE.

The Sharjah Strandings Response Program (SSRP), an initiative led by the Environment and Protected Areas Authority (EPAA) of Sharjah, was utilized in identifying stranded (beached) sea snakes along the Sharjah coasts. Through the SSRP's Strandings Response Network, stranded sea snakes were detected and reported to EPAA's team of stranding responders. In this study, all sea snakes that were used were deceased prior to being transported to the laboratory for further examination. However, live strandings were rescued and taken to the Breeding Center of Endangered Wildlife (BCEAW) for rehabilitation. For this particular study, we were interested in quantifying the levels of heavy metals in the tissues of sea snakes that died as a result of exposure to the November 2021 oil spill in Kalba (see Fig. 3) described by Yaghmour et al., [21]. It was observed that only two out of the examined oiled snake specimens displayed a moderate oil coverage of 50–75 %, whereas the remaining specimens exhibited a more severe coverage range of 75–100 %. Given the limited occurrence of stranded sea snakes observed along Sharjah's eastern coast outside this spill incident, we are using strandings collected from the nearby Arabian Gulf coast of Sharjah to compare the levels of heavy metals in sea snakes affected by the oil spill (see Fig. 3) against their non-oiled counterparts (see Fig. 1). A total of 24 dead strandings, collected between January 2018 and the end of 2021, were selected for this study. These specimens comprised 14 *Hydrophis lapemoides*, 6 *Hydrophis platurus*, and 4 *Hydrophis spiralis*.

Before collecting the stranded sea snakes, an external examination was conducted to document signs of harmful human interactions, record body measurements, identify species, stranding dates, and locations. Only specimens with mild decomposition were considered for inclusion in this study, while those showing significant decomposition were excluded. The snout-to-vent length (SVL) was deemed a reliable unit for body length, as tail lengths might be altered by injury or decomposition. The weight was also recorded. The subsequent step involved opening the coelomic cavity to conduct a gross examination of the internal organs. Tissues such as liver, muscle and fat were collected and individually wrapped in aluminum foil and stored at −20 °C prior to chemical analysis. Particular care was taken to prevent contamination of internal tissues in the case of oiled sea snakes.

### 2.3 Heavy metal analysis

Liver, muscle and fat tissues of the collected sea snakes were analyzed for metals including Arsenic (As), Cadmium (Cd), Chromium (Cr), Copper (Cu), Mercury (Hg), Manganese (Mn), Nickel (Ni), Lead (Pb), Selenium (Se), Aluminum (Al), Iron (Fe) and zinc (Zn). Tissue samples were digested with 10 mL ultrapure nitric acid (Honeywell-Fluka TraceSELECT), the mass of the sample was dependent on the tissue availability and it ranged from 0.1000 to 4.5 g (g). The samples were allowed to react at room temperature for at least 8 h prior to microwave acid digestion using CEM Corporation Microwave Accelerated Reaction System (MARS) microwave digestion unit. The microwave was set at 80 % power with 1600W max, with ramp time of 5.5 min & hold time of 4.5 min at 175**°**C. After digestion, samples were filtered using gravity filtration and diluted to 50 mL using deionised water. The diluted samples were quantitatively determined using an Inductively coupled plasma mass spectrometry (ICP-MS) detection (PerkinElmer Nexion ICP-MS 350X instrument). The calibration curves were developed using standard solutions for twelve metals ISO 17034 certified calibration standards obtained from Inorganic Ventures, USA prepared in the concentration range of 2–200 mg/L. Blank samples and Quality Control Standards were analyzed along with the batch of samples. Recoveries range from 73 to 95 %. Analysis was done with the support of Al Futtaim Element Materials Technology Dubai.

### 2.4 Data analysis

The significance between the concentration of heavy metals in oiled and non-oiled sea snakes was determined using Student's t-test, α = 0.05. Additionally, an analysis of covariances (ANCOVA) test was performed to investigate the differences in heavy metal concentrations between oiled and non-oiled sea snakes for the three tissue types. Before implementing the ANCOVA test, the normality of the data should be assessed. If the data departs from normality, a nonparametric approach should be followed, such as the Quade ANCOVA and Puri & Sen methods [27]. In this study, our data was found to be non-normally distributed. Consequently, the Quade ANCOVA method was implemented. This statistical test encompasses three main steps: (i) separately ranking the response variables and the covariates. (ii) Calculating the residual values from the linear regression formed by the ranked covariates, an independent variable, and the ranked response, which is, in this case, the heavy metal concentration. (iii) applying analysis of variance on the residuals to test the difference between the two groups of snakes, oiled and non-oiled. Since only two groups are to be compared, t-tests will be applied to the residuals obtained in step ii. This test is repeated for each tissue type's heavy metals residual values, ultimately leading to 39 independent t-tests as 13 heavy metals in three tissue types are investigated.

## 3 Results

In this study, we conducted an examination on 24 stranded sea snakes belonging to three species. Out of these, 14 were Arabian Gulf coral reef sea snakes (*Hydrophis lapemoides*), with 12 specimens sampled from the Arabian Gulf and 2 from the Gulf of Oman coast of Sharjah. Additionally, we sampled six yellow-bellied sea snakes (*Hydrophis platurus*), with 2 individuals from the Arabian Gulf and 4 from the Gulf of Oman coast of Sharjah. Furthermore, four yellow sea snakes (*Hydrophis spiralis*) were included in the study, all of which were collected from the Gulf of Oman. With the exception of one *Hydrophis spiralis* specimen, all the specimens from the Gulf of Oman had been exposed to oil at the time of their death while none of the specimens sampled from the Arabian Gulf were oiled. For more detailed information, this oil spill event is described in Yaghmour et al. [21].

All four yellow sea snakes in this study were collected from the eastern coast of Sharjah, along the coast of the city of Kalba. The sample comprised one non-oiled specimen and three oiled specimens. These sea snakes exhibited an average SVL of 102.0 ± 21.4 cm, with a range of 57–156 cm, and an average mass of 289.3 ± 86.5 g, ranging from 42 g to 736 g. Most of the metals analyzed in fat, liver and muscle were detected in 100 % of the oiled *Hydrophis spiralis* analyzed. Hg was found in 66.7 % of the fat tissue in oiled snakes and Cd was detected in 66.7 % of the muscle tissue. Fe was detected at the highest concentration in all samples where metals were detected, followed by Al and Zn. The lowest concentrations in fat and liver were reported for Hg, and Cd had the lowest concentration in muscle. Detailed information on the heavy metal concentrations in these yellow sea snakes is provided in Table 1.

**Table 1:** Percentage detection, mean, standard error, median and range of heavy metal residues detected in the fat, liver and muscle tissues sampled from Yellow Sea Snakes, *Hydrophis spiralis* from Kalba, Sharjah, United Arab Emirates.

	Specimen Group	Yellow Sea Snake *Hydrophis spiralis* [Ntot = 4; Noiled = 3; Nnon-oiled = 1]								
		Fat [N tot = 3; N oiled = 3 N non-oiled = 0]			Liver [N tot = 3; N oiled = 2; N non-oiled = 1]			Muscle [N tot = 4; N oiled = 3; N non-oiled = 1]		
		**%Det**	$\bar{x}$**± SE (mg/kg)**	**Med (R) (mg/kg)**	**%Det**	$\bar{x}$**± SE (mg/kg)**	**Med (R) (mg/kg)**	**%Det**	$\bar{x}$**± SE (mg/kg)**	**Med (R) (mg/kg)**
**Al**	**Total**	100.00	77.27 ± 39.93	56.75 (20.69–154.37)	100.00	84.89 ± 39.29	65.28 (28.79–160.60)	100.00	28.31 ± 11.28	21.43 (9.47–60.91)
	**Oiled**	100.00	77.27 ± 39.93	56.75 (20.69–154.37)	100.00	47.04 ± 18.25	(28.79–65.28)	100.00	17.44 ± 4.27	18.78 (9.47–24.08)
	**Non-Oiled**	N/A	–	–	100.00	160.60	–	100.00	60.91	–
**Se**	**Total**	100.00	0.62 ± 0.16	0.50 (0.42–0.93)	100.00	1.12 ± 0.23	0.94 (0.84–1.57)	75.00	0.32 ± 0.12	0.37 (BD-0.55)
	**Oiled**	100.00	0.62 ± 0.16	0.50 (0.42–0.93)	100.00	1.25 ± 0.32	(0.94–1.57)	100.00	0.43 ± 0.07	0.43 (0.31–0.55)
	**Non-Oiled**	N/a	–	–	100.00	0.84	–	0.00	BD	–
**Cd**	**Total**	100.00	0.63 ± 0.31	0.49 (0.17–1.22)	100.00	0.51 ± 0.15	0.54 (0.23–0.75)	75.00	0.05 ± 0.03	0.04 (BD-0.14)
	**Oiled**	100.00	0.63 ± 0.31	0.49 (0.17–1.22)	100.00	0.64 ± 0.10	(0.54–0.75)	66.67	0.06 ± 0.04	0.03 (0.00–0.14)
	**Non-Oiled**	N/A	–	–	100.00	0.23	–	100.00	0.04	–
**Hg**	**Total**	66.67	0.04 ± 0.03	0.03 (BD-0.09)	100.00	0.10 ± 0.05	0.08 (0.03–0.20)	75.00	0.11 ± 0.07	0.07 (BD-0.32)
	**Oiled**	66.67	0.04 ± 0.03	0.03 (BD-0.09)	100.00	0.14 ± 0.06	(0.08–0.20)	100.00	0.15 ± 0.09	0.10 (0.04–0.32)
	**Non-Oiled**	N/A	–	–	100.00	0.03	–	0.00	BD	–
**Pb**	**Total**	100.00	1.79 ± 1.38	0.61 (0.22–4.55)	100.00	0.51 ± 0.32	0.24 (0.14–1.14)	75.00	0.16 ± 0.07	0.16 (BD-0.34)
	**Oiled**	100.00	1.79 ± 1.38	0.61 (0.22–4.55)	100.00	0.69 ± 0.45	(0.24–1.14)	100.00	0.22 ± 0.06	0.18 (0.14–0.34)
	**Non-Oiled**	N/A	–	–	100.00	0.14	–	0.00	BD	–
**Cr**	**Total**	100.00	1.20 ± 0.78	0.66 (0.20–2.74)	100.00	0.35 ± 0.11	0.25 (0.24–0.57)	100.00	0.34 ± 0.13	0.32 (0.10–0.64)
	**Oiled**	100.00	1.20 ± 0.78	0.66 (0.20–2.74)	100.00	0.41 ± 0.16	(0.25–0.57)	100.00	0.29 ± 0.17	0.13 (0.10–0.64)
	**Non-Oiled**	N/A	–	–	100.00	0.24	–	100.00	0.5	–
**Mn**	**Total**	100.00	2.37 ± 1.48	1.34 (0.47–5.28)	100.00	0.82 ± 0.05	0.83 (0.72–0.90)	100.00	0.36 ± 0.17	0.24 (0.10–0.85)
	**Oiled**	100.00	2.37 ± 1.48	1.34 (0.47–5.28)	100.00	0.86 ± 0.04	(0.83–0.90)	100.00	0.20 ± 0.08	0.13 (0.10–0.36)
	**Non-Oiled**	N/A	–	–	100.00	0.72	–	100.00	0.85	–
**Fe**	**Total**	100.00	227.03 ± 77.05	233.00 (90.70–357.39)	100.00	230.75 ± 50.06	220.44 (149.66–322.15)	100.00	45.38 ± 14.90	32.38 (27.27–89.50)
	**Oiled**	100.00	227.03 ± 77.05	233.00 (90.70–357.39)	100.00	185.05 ± 35.39	(149.66–220.44)	100.00	30.68 ± 3.38	27.33 (27.27–37.43)
	**Non-Oiled**	N/A	–	–	100.00	322.15	–	100.00	89.5	–
**Ni**	**Total**	100.00	6.49 ± 3.75	5.21 (0.73–13.54)	100.00	1.96 ± 1.11	1.43 (0.36–4.09)	100.00	0.92 ± 0.36	1.03 (BD-1.62)
	**Oiled**	100.00	6.49 ± 3.75	5.21 (0.73–13.54)	100.00	2.76 ± 1.33	(1.43–4.09)	100.00	0.76 ± 0.33	0.70 (0.22–1.36)
	**Non-Oiled**	N/A	–	–	100.00	0.36	–	100.00	1.62	–
**Cu**	**Total**	100.00	18.26 ± 10.55	11.78 (4.12–38.88)	100.00	10.03 ± 3.46	8.52 (4.94–16.63)	100.00	4.04 ± 1.30	4.14 (0.87–7.03)
	**Oiled**	100.00	18.26 ± 10.55	11.78 (4.12–38.88)	100.00	12.57 ± 4.05	(8.52–16.63)	100.00	5.10 ± 1.07	4.93 (3.35–7.03)
	**Non-Oiled**	N/A	–	–	100.00	4.94	–	100.00	0.87	–
**Zn**	**Total**	100.00	49.35 ± 26.76	30.92 (15.04–102.08)	100.00	47.57 ± 9.68	41.03 (35.06–66.63)	100.00	20.97 ± 4.19	20.83 (11.43–30.79)
	**Oiled**	100.00	49.35 ± 26.76	30.92 (15.04–102.08)	100.00	53.83 ± 12.80	(41.03–66.63)	100.00	24.15 ± 3.85	24.22 (17.44–30.79)
	**Non-Oiled**	N/A	–	–	100.00	35.06	–	100.00	11.43	–
**As**	**Total**	100.00	5.14 ± 3.28	2.07 (1.67–11.69)	100.00	6.64 ± 5.58	1.41 (0.72–17.81)	100.00	5.43 ± 4.54	1.14 (0.39–19.04)
	**Oiled**	100.00	5.14 ± 3.28	2.07 (1.67–11.69)	100.00	9.61 ± 8.20	(1.41–17.81)	100.00	7.11 ± 5.97	1.33 (0.95–19.04)
	**Non-Oiled**	N/A	–	–	100.00	0.72	–	100.00	0.39	–

**N/A:** not applicable; **% Det:** percent detection among all sampled specimens; $\bar{x}$**:** average mean; **SE:** standard error; **Med:** median concentration among all sampled specimens; **R:** range of concentrations; **BD:** below detection.

Four oiled yellow-bellied sea snakes were collected from the eastern coast of Sharjah, along the coast of the city of Kalba, while two non-oiled specimens were collected from the western coast of Sharjah, along the coast of the city of Sharjah. The sample consisted of three females and three males. The mean SVL of 41.7 ± 5.26 cm, ranging from 18.4 to 53.2 cm, and the mean mass was 41.7 ± 5.26 g, ranging from 22.0 to 148.0 g. Metals analyzed in fat, liver and muscle showed that the major metals found were Al, Fe and Zn consistently. In fat, Al was at the highest concentration. Fe was above the concentration of all other metals in liver, and Zn was found at highest concentration in muscle. Hg and Cd, were consistently found at the lowest concentrations in all studied tissues. Al, Pb, Cr, Mn, Fe, Cu, Zn and As were detected in 100 % of both oiled and non-oiled samples of *Hydrophis platurus*. Se was detected in 100 % of liver, muscle and non-oiled fat, but in 75 % of oiled fat. Interestingly, Hg was found in 25 % of the oiled fat and muscle tissues, but not in the non-oiled. In the case of Ni, it was found mostly in oiled tissues. The concentrations of heavy metals in yellow-bellied sea snakes are detailed in Table 2.

**Table 2:** Percentage detection, mean, standard error, median and range of heavy metal residues detected in the fat, liver and muscle tissues sampled from Yellow-Bellied Sea Snakes, *Hydrophis platurus* from Sharjah, United Arab Emirates (Sharjah and Kalba).

	Specimen Group	Yellow-Bellied Sea Snake *Hydrophis platurus* [Ntot = 6; Noiled = 4; Nnon-oiled = 2]								
		Fat [N tot = 6; N oiled = 4; N non-oiled = 2]			Liver [N tot = 5; N oiled = 4; N non-oiled = 1]			Muscle [N tot = 6; N oiled = 4; N non-oiled = 2]		
		%Det	$\bar{x}$ ± SE (ppm)	Med (R) (ppm)	%Det	$\bar{x}$ ± SE (ppm)	Med (R) (ppm)	%Det	$\bar{x}$ ± SE (ppm)	Med (R) (ppm)
**Al**	**Total**	100.00	78.53 ± 20.38	86.03 (3.70–121.41)	100.00	40.33 ± 20.24	19.56 (6.90–139.26)	100.00	20.77 ± 2.97	22.94 (8.66–28.65)
	**Oiled**	100.00	97.23 ± 10.43	96.39 (74.74–121.41)	100.00	48.99 ± 30.09	19.56 (17.57–139.26)	100.00	22.70 ± 2.64	22.94 (16.27–28.65)
	**Non-Oiled**	100.00	3.70	–	100.00	23.02	(6.90–39.13)	100.00	16.92	(8.66–25.18)
**Se**	**Total**	80.00	0.30 ± 0.09	0.30 (BD-0.50)	100.00	1.30 ± 0.18	1.24 (0.86–2.03)	100.00	0.53 ± 0.06	0.53 (0.31–0.68)
	**Oiled**	75.00	0.26 ± 0.09	0.29 (BD-0.44)	100.00	1.05 ± 0.11	1.02 (0.86–1.30)	100.00	0.59 ± 0.05	0.62 (0.45–0.68)
	**Non-Oiled**	100.00	0.50	–	100.00	1.79	(1.55–2.03)	100.00	0.4	(0.31–0.49)
**Cd**	**Total**	100.00	0.11 ± 0.03	0.09 (0.03–0.24)	100.00	0.78 ± 0.14	0.79 (0.28–1.36)	50.00	0.01 ± 0.01	BD (BD-0.03)
	**Oiled**	100.00	0.12 ± 0.04	0.10 (0.03–0.24)	100.00	0.63 ± 0.13	0.69 (0.28–0.86)	75.00	0.02 ± 0.01	0.03 (BD-0.03)
	**Non-Oiled**	100.00	0.08	–	100.00	1.09	(0.81–1.36)	0.00	BD	–
**Hg**	**Total**	20.00	–	–	33.33	0.04 ± 0.04	BD (BD-0.24)	16.67	0.01 ± 0.01	BD (BD-0.04)
	**Oiled**	25.00	0.01 ± 0.01	BD (BD-0.02)	0.00	BD	–	25.00	0.01 ± 0.01	BD (BD-0.04)
	**Non-Oiled**	0.00	BD	–	100.00	0.13	(0.02–0.24)	0.00	BD	–
**Pb**	**Total**	100.00	0.77 ± 0.18	0.85 (0.07–1.18)	100.00	0.57 ± 0.22	0.38 (0.15–1.54)	100.00	0.45 ± 0.14	0.30 (0.27–1.12)
	**Oiled**	100.00	0.95 ± 0.08	0.88 (0.84–1.18)	100.00	0.63 ± 0.31	0.38 (0.20–1.54)	100.00	0.31 ± 0.04	0.28 (0.27–0.42)
	**Non-Oiled**	100.00	0.07	–	100.00	0.44	(0.15–0.73)	100.00	0.71	(0.30–1.12)
**Cr**	**Total**	100.00	0.38 ± 0.08	0.36 (0.14–0.59)	100.00	0.44 ± 0.20	0.15 (0.11–1.19)	100.00	0.35 ± 0.17	0.20 (0.15–1.19)
	**Oiled**	100.00	0.44 ± 0.07	0.44 (0.31–0.59)	100.00	0.34 ± 0.19	0.15 (0.14–0.91)	100.00	0.19 ± 0.02	0.19 (0.15–0.22)
	**Non-Oiled**	100.00	0.14	–	100.00	0.65	(0.11–1.19)	100.00	0.69	(0.20–1.19)
**Mn**	**Total**	100.00	0.62 ± 0.19	0.57 (0.20–1.28)	100.00	0.74 ± 0.22	0.71 (0.15–1.59)	100.00	0.35 ± 0.11	0.26 (0.13–0.87)
	**Oiled**	100.00	0.73 ± 0.20	0.64 (0.35–1.28)	100.00	0.46 ± 0.15	0.47 (0.15–0.74)	100.00	0.20 ± 0.05	0.18 (0.13–0.34)
	**Non-Oiled**	100.00	0.2	–	100.00	1.31	(1.03–1.59)	100.00	0.65	(0.44–0.87)
**Fe**	**Total**	100.00	55.50 ± 11.40	45.52 (33.09–84.33)	100.00	503.34 ± 242.89	319.98 (60.67–1661.37)	100.00	31.97 ± 10.51	24.39 (15.70–83.55)
	**Oiled**	100.00	61.10 ± 12.82	63.45 (33.17–84.33)	100.00	234.25 ± 105.09	171.06 (60.67–534.18)	100.00	20.10 ± 2.49	19.91 (15.70–24.88)
	**Non-Oiled**	100.00	33.09	–	100.00	1041.52	(421.66–1661.37)	100.00	55.7	(27.86–83.55)
**Ni**	**Total**	80.00	1.64 ± 0.64	1.87 (BD-3.06)	83.33	5.60 ± 4.48	1.47 (BD-27.95)	83.33	1.44 ± 0.45	1.19 (BD-3.32)
	**Oiled**	100.00	2.05 ± 0.64	2.41 (0.32–3.06)	100.00	8.10 ± 6.63	1.94 (0.55–27.95)	100.00	1.68 ± 0.55	1.19 (1.02–3.32)
	**Non-Oiled**	0.00	BD	–	50.00	0.61	(BD-1.22)	50.00	0.97	(0.00–1.94)
**Cu**	**Total**	100.00	10.39 ± 2.68	10.52 (1.96–18.71)	100.00	13.84 ± 5.43	6.73 (6.16–39.34)	100.00	9.01 ± 2.69	6.87 (4.66–22.33)
	**Oiled**	100.00	12.49 ± 2.13	11.06 (9.15–18.71)	100.00	14.68 ± 8.22	6.62 (6.16–39.34)	100.00	6.36 ± 0.57	6.87 (4.66–7.02)
	**Non-Oiled**	100.00	1.96	–	100.00	12.15	(6.43–17.87)	100.00	14.31	(6.28–22.33)
**Zn**	**Total**	100.00	29.88 ± 3.16	32.12 (17.47–34.55)	100.00	59.50 ± 16.94	49.00 (19.08–123.15)	100.00	34.10 ± 4.66	32.27 (22.91–52.71)
	**Oiled**	100.00	32.98 ± 0.77	33.05 (31.27–34.55)	100.00	50.63 ± 24.45	30.14 (19.08–123.15)	100.00	27.85 ± 3.00	26.20 (22.91–36.12)
	**Non-Oiled**	100.00	17.47	–	100.00	77.23	(61.58–92.88)	100.00	46.60	(40.50–52.71)
**As**	**Total**	100.00	1.86 ± 0.21	1.71 (1.37–2.40)	100.00	1.20 ± 0.08	1.25 (0.84–1.40)	100.00	1.12 ± 0.07	1.12 (0.85–1.38)
	**Oiled**	100.00	1.99 ± 0.22	2.02 (1.50–2.40)	100.00	1.17 ± 0.12	1.25 (0.84–1.34)	100.00	1.04 ± 0.08	1.05 (0.85–1.22)
	**Non-Oiled**	100.00	1.37	–	100.00	1.25	(1.11–1.40)	100.00	1.28	(1.18–1.38)

**% Det:** percent detection among all sampled specimens; $\bar{x}$**:** average mean; **SE:** standard error; **Med:** median concentration among all sampled specimens; **R:** range of concentrations; **BD:** below detection.

Two oiled Arabian Gulf Coral Reef Sea Snakes were collected from the eastern coast of Sharjah, along the coast of the city of Kalba, while 12 non-oiled specimens were collected from the western coast of Sharjah, along the coast of the city of Sharjah. The mean SVL of 78.73 ± 3.52 cm, ranging from 61.5 to 84.2 cm, and the mean mass was 41.7 ± 5.26 g, ranging from 49.0 to 394.0 g. The analysis of Metals in fat, liver and muscle of *Hydrophis lapemoides* resulted in the same trend found in the other species where the highest concentrations were reported for Fe, Al and Zn, in decreasing order. As in the other samples, the same two metals Hg and Cd were consistently found at the lowest concentrations. Al, Pb, Cr, Mn, Fe, Cu, Zn and As were detected in 100 % of both oiled and non-oiled samples. Se was detected in 100 % of liver, muscle and oiled fat, but in 70 % of non-oiled fat. Cd was detected mainly in oiled samples and Hg was detected in all liver and muscle, but only in 30 % of non-oiled fat tissues. As in the case of *Hydrophis platurus,* Ni was found mostly in oiled tissues. The concentrations of heavy metals in Arabian Gulf Coral Reef Sea Snakes are detailed in Table 3.

**Table 3:** Percentage detection, mean, standard error, median and range of heavy metal residues detected in the fat, liver and muscle tissues sampled from Arabian Gulf coral reef sea snakes, *Hydrophis lapemoides* from Sharjah, United Arab Emirates (Sharjah and Kalba).

	Specimen Group	Arabian Gulf Sea Snake *Hydrophis lapemoides* [Ntot = 14; Noiled = 2; Nnon-oiled = 12]								
		Fat [N tot = 12; N oiled = 2; N non-oiled = 10]			Liver [N tot = 11; N oiled = 1; N non-oiled = 10]			Muscle [N tot = 11; N oiled = 2; N non-oiled = 9]		
		**%Det**	$\bar{x}$**± SE (ppm)**	**Med (R) (ppm)**	**%Det**	$\bar{x}$**± SE (ppm)**	**Med (R) (ppm)**	**%Det**	$\bar{x}$**± SE (ppm)**	**Med (R) (ppm)**
**Al**	**Total**	100.00	32.06 ± 8.50	21.64 (3.65–106.14)	100.00	38.97 ± 14.56	19.77 (1.95–156.77)	100.00	16.26 ± 3.00	17.91 (1.49–29.40)
	**Oiled**	100.00	74.45	(42.77–106.14)	100.00	119.33	N/A	100.00	22.31	(18.04–26.58)
	**Non-Oiled**	100.00	23.58 ± 5.97	18.96 (3.65–55.34)	100.00	31.67 ± 13.80	18.47 (1.95–156.77)	100.00	14.92 ± 3.46	16.80 (1.49–29.40)
**Se**	**Total**	75.00	0.28 ± 0.06	0.34 (BD-0.52)	100.00	1.72 ± 0.12	1.57 (1.32–2.53)	100.00	0.74 ± 0.12	0.57 (0.39–1.61)
	**Oiled**	100.00	0.42	(0.33–0.51)	100.00	1.32	N/A	100.00	0.44	(0.39–0.48)
	**Non-Oiled**	70.00	0.27 ± 0.07	0.32 (BD-0.52)	100.00	1.76 ± 0.12	1.61 (1.34–2.53)	100.00	0.80 ± 0.14	0.59 (0.41–1.61)
**Cd**	**Total**	25.00	0.03 ± 0.02	BD (BD-0.28)	100.00	0.27 ± 0.14	0.07 (0.03–1.66)	27.27	0.01 ± 0.01	BD (BD-0.08)
	**Oiled**	100.00	0.16	(0.04–0.28)	100.00	0.55	N/A	100.00	0.04	(0.02–0.07)
	**Non-Oiled**	10.00	0.00 ± 0.00	BD (BD-0.03)	100.00	0.24 ± 0.15	0.06 (0.03–1.66)	11.11	0.01 ± 0.01	BD (BD-0.08)
**Hg**	**Total**	25.00	0.00 ± 0.00	BD (BD-0.05)	100.00	0.08 ± 0.03	0.04 (BD- 0.32)	100.00	0.15 ± 0.06	0.08 (0.03–0.70)
	**Oiled**	0.00	BD	–	100.00	0.03	N/A	100.00	0.03	(0.03–0.04)
	**Non-Oiled**	30.00	0.01 ± 0.01	BD (BD-0.05)	100.00	0.08 ± 0.03	0.05 (BD-0.32)	100.00	0.18 ± 0.07	0.11 (0.03–0.70)
**Pb**	**Total**	100.00	0.33 ± 0.10	0.24 (BD-1.28)	90.90	0.20 ± 0.04	0.18 (BD-0.43)	81.81	0.24 ± 0.06	0.21 (BD-0.60)
	**Oiled**	100.00	0.46	(0.45–0.47)	100	0.43	N/A	100.00	0.25	(0.20–0.30)
	**Non-Oiled**	100.00	0.31 ± 0.12	0.21 (0.04–1.28)	90.00	0.17 ± 0.04	0.16 (BD-0.43)	77.78	0.24 ± 0.07	0.21 (BD-0.60)
**Cr**	**Total**	100.00	0.43 ± 0.12	0.29 (0.10–1.55)	100.00	0.38 ± 0.16	0.15 (0.06–1.82)	100.00	0.26 ± 0.06	0.19 (BD-0.69)
	**Oiled**	100.00	1.2	(0.85–1.55)	100.00	1.82	N/A	100.00	0.4	(0.11–0.69)
	**Non-Oiled**	100.00	0.28 ± 0.05	0.22 (0.10–0.54)	100.00	0.25 ± 0.10	0.11 (0.06–1.18)	100.00	0.23 ± 0.05	0.19 (0.04–0.55)
**Mn**	**Total**	100.00	0.72 ± 0.28	0.42 (0.13–3.72)	100.00	1.09 ± 0.36	0.69 (0.46–4.99)	100.00	0.39 ± 0.07	0.38 (0.09–0.88)
	**Oiled**	100.00	2.27	(0.81–3.72)	100.00	4.99	N/A	100.00	0.4	(0.26–0.55)
	**Non-Oiled**	100.00	0.41 ± 0.07	0.36 (0.13–0.80)	100.00	0.74 ± 0.06	0.65 (0.46–1.04)	100.00	0.39 ± 0.09	0.38 (0.09–0.88)
**Fe**	**Total**	100.00	73.21 ± 23.61	45.23 (18.59–313.11)	100.00	686.24 ± 205.12	469.37 (96.66–2397.55)	100.00	29.78 ± 3.57	30.95 (9.20–47.39)
	**Oiled**	100.00	200.33	(87.55–313.11)	100.00	420.95	N/A	100.00	39.17	(30.95–47.39)
	**Non-Oiled**	100.00	47.78 ± 10.21	31.56 (18.59–113.62)	100.00	710.36 ± 223.14	478.02 (96.66–2397.55)	100.00	27.69 ± 3.82	28.05 (9.20–41.09)
**Ni**	**Total**	25.00	2.33 ± 0.92	0.87 (BD-10.03)	72.73	2.19 ± 1.06	0.82 (BD-12.84)	72.73	2.73 ± 1.32	0.88 (BD-14.97)
	**Oiled**	100.00	6.36	(2.68–10.03)	100.00	12.84	N/A	100.00	2.61	(2.54–2.67)
	**Non-Oiled**	11.11	1.53 ± 0.71	0.35 (BD-6.58)	70.00	1.22 ± 0.48	0.59 (BD-4.95)	66.67	2.75 ± 1.63	0.79 (BD-14.97)
**Cu**	**Total**	100.00	5.86 ± 1.75	4.12 (0.68–22.64)	100.00	6.73 ± 0.76	6.43 (2.19–11.18)	100.00	3.97 ± 0.67	4.20 (0.66–7.12)
	**Oiled**	100.00	9.32	(8.63–10.01)	100.00	8.07	N/A	100.00	5.32	(3.93–6.72)
	**Non-Oiled**	100.00	5.16 ± 2.04	3.55 (0.68–22.64)	100.00	6.61 ± 0.83	6.24 (2.19–11.18)	100.00	3.67 ± 0.75	4.20 (0.66–7.12)
**Zn**	**Total**	100.00	19.91 ± 2.59	18.40 (6.65–39.75)	100.00	31.82 ± 2.46	33.93 (17.41–48.82)	100.00	41.51 ± 9.04	36.08 (17.24–125.91)
	**Oiled**	100.00	28.53	(26.53–30.53)	100.00	30.13	N/A	100.00	33.96	(17.24–50.69)
	**Non-Oiled**	100.00	18.19 ± 2.79	17.42 (6.65–39.75)	100.00	31.98 ± 2.69	34.33 (17.41–48.82)	100.00	43.19 ± 10.73	36.08 (18.53–125.91)
**As**	**Total**	100.00	2.57 ± 0.42	2.48 (0.58–5.27)	100.00	6.59 ± 2.08	2.83 (0.53–21.25)	100.00	10.64 ± 2.59	12.81 (0.51–24.52)
	**Oiled**	100.00	2.96	(2.47–3.44)	100.00	21.25	N/A	100.00	10.2	(1.22–19.17)
	**Non-Oiled**	100.00	2.49 ± 0.50	1.98 (0.58–5.27)	100.00	5.25 ± 1.75	2.16 (0.53–15.77)	100.00	10.73 ± 2.83	12.81 (0.51–24.52)

**% Det:** percent detection among all sampled specimens; $\bar{x}$**:** average mean; **SE:** standard error; **Med:** median concentration among all sampled specimens; **R:** range of concentrations; **BD:** below detection.

The concentrations of various heavy metals in muscle, fat, and liver tissues of oiled and non-oiled sea snakes were compared using a students t-test. In muscle tissue, no significant difference was observed between the concentration of Al (t = −0.31069, p = 0.759416), Se (t = 1.06372, p = 0.300791), Cd (t = −1.85312, p = 0.079461), Hg (t = 0.96903, p = 0.344705), Pb (t = 0.27215, p = 0.78844), Cr (t = 0.51605, p = 0.611776), Fe (t = 1.09208, p = 0.288454), Ni (t = 0.53691, p = 0.597559), Cu (t = −0.2489, p = 0.806105), Zn (t = 1.2845, p = 0.214409) and As (t = 0.87726, p = 0.391305) in oiled and non-oiled sea snakes. However, Mn was observed at significantly greater concentrations (t = 2.1391, p = 0.045627) in non-oiled sea snakes.

In fat tissue, no significant difference was observed between the concentration of Se (t = −1.14947, p = 0.265409), Hg (t = −0.7036, p = 0.490683), Pb (t = −2.02241, p = 0.058251), Ni (t = −2.03125, p = 0.057264) and Cu (t = −0.79311, p = 0.438044). However, Al (t = −4.55523, p = 0.000245), Cd (t = −2.55929, p = 0.019713), Cr (t = −2.42709, p = 0.025941), Mn (t = −2.36413, p = 0.029517), Fe (t = −2.67243, p = 0.015534), Cu (t = −2.39429, p = 0.02775), and Zn (t = −2.42109, p = 0.026264) were observed at significantly greater concentrations in non-oiled sea snakes.

Finally, in liver tissue, no significant difference was observed between the concentration of Al (t = −0.76894, p = 0.451384), Cd (t = −1.24665, p = 0.227676), Hg (t = 1.06144, p = 0.301796), Cr (t = −1.21083, p = 0.240808), Mn (t = −0.89086, p = 0.384149), Fe (t = 1.74647, p = 0.096877), Cu (t = −1.67761, p = 0.109797), Zn (t = −0.8323, p = 0.41558) and As (t = −0.67009, p = 0.510867) in oiled and non-oiled sea snakes. In liver, Se (t = 2.98379, p = 0.007631) was observed at a significantly greater concentration in non-oiled sea snakes, while Pb (t = −2.64396, p = 0.016007) and Ni (t = −2.31249, p = 0.032119) were observed at significantly greater concentrations in oiled sea snakes.

**Fig. 4:**
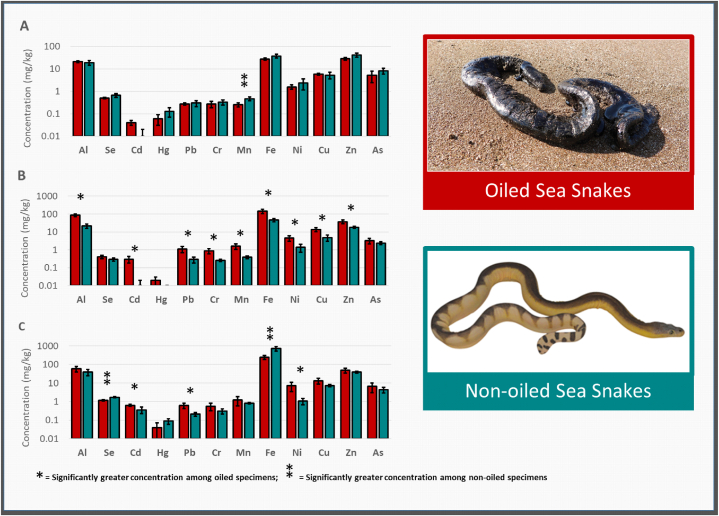
Logarithmic histograms showing a **c**omparison of heavy metal concentrations in the tissues of oiled and non-oiled sea snakes **[A-C]**. Concentration of heavy metals in muscle **[A]**, fat **[B]** and liver **[C]** tissues. A single asterisk (*) signifies significantly greater concentration among oiled specimens. A double asterisk () signifies significantly greater concentration among non-oiled specimens according to nonparametric ANCOVA multiple tests.

To understand how heavy metal concentrations differ between oiled and non-oiled snakes in each tissue type while considering the covariances for mass and SVL, analysis of covariance (ANCOVA) test was applied for each heavy metal concentration. Prior to applying ANCOVA, the normality of the data distribution was evaluated using the Shapiro-Wilk test; the results revealed that the data were not normally distributed. Accordingly, a nonparametric ANCOVA approach was followed, as explained in the methodology section. The p-values were calculated and reported in Table 1. As illustrated in Table 4, the concentration of most of the measured heavy metals, including AI, Pb, Cr, Mn, Fe, Ni, Cu, Zn, Cd, and total heavy metals (Total_HM), are significantly higher in oiled snakes compared to non-oiled snakes. In the liver tissue, Se and Fe were significantly lower in oiled than non-oiled snakes, while Se and Ni were significantly higher. The remaining heavy metals show non-significant differences among the two groups of snakes. Interestingly, no significant difference was found among all heavy metal concentrations in the muscle tissues of oiled and non-oiled snakes except for Mn, which was significantly lower in oiled snakes than non-oiled snakes. These results are summarized in Table .4 and Fig. 4.

**Table 4:** Nonparametric ANCOVA multiple tests results comparing heavy metal concentrations in oiled versus non-oiled sea snakes.

Heavy metal	Difference ratio (oiled – not oiled)			p-value		
	Fat	Liver	Muscle	Fat	Liver	Muscle
**Al**	4.567	1.805	0.561	0.0002^a^	0.088	0.581
**Se**	0.773	−4.249	−0.823	0.449	0.0005**⁑**	0.421
**Cd**	5.048	2.541	1.229	0.000^a^	0.020^a^	0.234
**Hg**	1.198	−1.085	−0.825	0.246	0.292	0.419
**Pb**	3.609	3.021	−0.645	0.002^a^	0.007^a^	0.526
**Cr**	2.880	1.988	−1.422	0.010^a^	0.062	0.171
**Mn**	3.806	−0.296	−2.630	0.001^a^	0.770	0.016**⁑**
**Fe**	3.691	−2.153	−1.697	0.002^a^	0.045**⁑**	0.106
**Ni**	3.122	2.986	0.713	0.006^a^	0.008^a^	0.484
**Cu**	3.598	1.615	0.700	0.002^a^	0.124	0.492
**Zn**	3.241	0.548	−2.480	0.005^a^	0.590	0.226
**As**	0.956	0.087	−0.208	0.352	0.931	0.838
**Total HM**	4.493	−1.416	−0.972	0.0003^a^	0.174	0.343

⁑ significantly greater concentration among non-oiled specimens.

a Significantly greater concentration among oiled specimens.

## 4 Discussion

In this study, we investigated the concentrations of heavy metals in the liver, muscle, and fat tissues of stranded sea snakes from Sharjah, UAE. Our samples comprised 24 specimens, including14 *Hydrophis lapemoides*, 6 *Hydrophis platurus* and 4 *Hydrophis spiralis*. This study marks the first assessment of heavy metals concentrations in any of these sea snake species, providing valuable insights into their exposure to environmental pollutants. The results of our analysis revealed noteworthy trends, with significantly higher concentrations of Al, Cd, Pb, Cr, Mn, Fe, Ni, Cu and Zn observed in sea snakes that were mired in oil. Interestingly, most of these significant differences were found in the fat tissue, indicating a potential association between oil exposure and the accumulation of heavy metals in this particular tissue. Examinations of the sea snake specimens mired in oil suggests that the viscosity and stickiness of the oil were the primary cause of snake mortalities [21].

Previous studies discussed the limitations in the conservation of sea snakes due to the lack of knowledge on their general demography, breeding cycles and taxonomy [28]. Furthermore, limited data is available on the impact of petroleum to sea snakes [29] but it is suggested that, due to their feeding patterns, exposure to contaminated sediments and water, they are vulnerable to oil spills [[21], [30]]. In general, reptiles such as turtles, crocodiles, alligators and sea snakes are all vulnerable to oil exposure at the surface or shallow waters and the risk of its consequences [[31], [32]]. The route of exposure for marine fauna to the metals is uncertain [26], though Goiran et al. [22], suggested that the main route for sea snakes is through ingestion of contaminated prey. Sea snakes possess a critical physiological characteristic of relying on the regulated movement of water and salts through their skin which allows for their survival in the marine environment [33]. This raises the possibility that these toxins might be directly absorbed through the skin. Goiran et al. [22], also suggested that sea snakes could potentially absorb heavy metals directly from the water due to their high surface area-to-volume ratio and substantial rates of gas exchange across their skin [[33], [34]]. It should be noted that research on the dermal exposure of reptiles to toxins is limited, which limits our ability to make comparisons with other uptake routes [34]. However, it is suggested that if a reptile's epidermal tissue possesses a high lipid content, which serves to prevent water loss, it may consequently facilitate increased absorption of lipophilic substances such as oils [34]. This is supported by a study conducted by Sereshk [24] who observed elevated levels of low molecular-weight polycyclic aromatic hydrocarbons (PAHs) in the skin of two sea snake species inhabiting the Arabian Gulf. Indeed, cutaneous exposure is recognized as a potential mechanism of toxin exposure in terrestrial snakes [25], and it is reasonable to consider that marine snakes may also experience transdermal absorption of contaminants when directly exposed during oil spills, provided they survive the immediate effects of oil intoxication and dermal damage. This aspect adds to the complexities of understanding the potential impacts of oil pollution on marine reptiles, necessitating further investigation into their response to and recovery from such exposure events.

Oil spills are recognized as a significant source of heavy metal exposure. A review article by Freije [35], reported on the environmental repercussions of the 1991 Gulf War, focused particularly on marine ecosystems. This study evaluated available published data pertaining to the presence of heavy metals, trace elements, and petroleum hydrocarbon pollution in soil, water, and various organisms. Another study assessed metal concentrations in oysters and gastropods prior to and after the 1991 Gulf War Oil Spill [36]. This study aligns with our findings, as it reports that the concentrations of Cu, Ni and Zn increased significantly after the spill. However, their observation of a slight decrease in the concentration of Pb conflicts with our study [36]. Similarly, a study examining bivalves collected from the Saudi coast revealed that specimens gathered in proximity to Kuwaiti waters exhibited a notably higher increase in heavy metal concentrations after the Gulf War spill when compared to prior assessments conducted in 1984. This observation strongly implies a direct impact of the oil spill on metal accumulation [37]. A study assessing the Niger Delta region in Nigeria, which is known for crude oil activities aligned with our study when reporting the presence of metals such as Mn, Fe, Cu, Zn, Pb, Ni, Cd and Cr in the oil [38].

A study by Osuji and Onojake [39], also aligns with our findings, reporting Ni, Cu, Cd, and Pb in the soil collected from Ebocha-8 Oil-Spill-Polluted Site in Niger. Furthermore, Mn, Cu, Ni, Cr, Al and Fe have been associated with MC252 oil from the Deepwater Horizon oil spill [26]. The study reported that whale skin exposed to oil from the Deepwater Horizon oil spill had a decline in the concentration of Mn, Cu, Ni, Cr, Al and Fe two years after the spill and an increase in Pb and Zn during that time [26]. To investigate the relationship between oil and heavy metals, Bruno et al. [40] tested heavy metals in a region in Rio de Janeiro, Brazil that is influenced by ports, increased urbanization, and oil platforms. Results indicated concentrations of Mn, Fe, Co, Ni, Cu, Zn, Cd, Hg, and Pb were higher in the kidney than in the muscle samples of green sea turtles. On the other hand, Al and As were higher in the muscle tissue, suggesting that both elements have inefficient detoxification.

## 5 Conclusion

As marine reptiles, sea snakes occupy a unique niche in the coastal food webs, and any disruption in their population dynamics may have cascading effects on the marine ecosystem. Further understanding the ecological significance of sea snakes in the UAE is essential for implementing effective conservation strategies to protect both the species and the marine environments they inhabit. Effective sea snake conservation also relies on our knowledge of the natural and anthropogenic threats that sea snakes face. In addition to heavy metal exposure, sea snakes face a range of environmental threats, including habitat degradation due to dredging, coastal development and pollution from various sources [4]. Climate change-related impacts, such as rising sea temperatures and ocean acidification, may further exacerbate the vulnerability of sea snakes and other marine species [4]. The results of this study support conservation efforts through the development of targeted management strategies to protect sea snake populations and the broader marine environment. However, given the number of samples, the utilization of different species, the varying sampling locations, the limited knowledge on the source of the oil spill, and the lack of previous baseline data on heavy metals in sea snakes, the results of this study should be interpreted with caution. Continued research in this area is essential to better understand the impacts and establish more comprehensive baseline data. Additionally, further sampling is needed to better understand the interspecific distinctions of heavy metal concentrations between different species of sea snakes as well as intraspecific distinctions between different age classes and coastlines. Further studies should also investigate the level of persistent organic pollutants in sea snakes as well as the effect of heavy metals and other pollutants on sea snake health and physiology.

## Data availability

Data associated with the study has not been deposited into a publicly available repository. Data are available from the corresponding author on reasonable request.

## CRediT authorship contribution statement

**Fadi Yaghmour:** Writing – review & editing, Writing – original draft, Visualization, Methodology, Investigation, Formal analysis, Data curation, Conceptualization. **Fatin Samara:** Writing – review & editing, Writing – original draft, Methodology, Investigation, Funding acquisition, Formal analysis, Data curation. **Yehya El Sayed:** Writing – review & editing, Writing – original draft, Funding acquisition, Formal analysis. **Areej Mohammed:** Writing – review & editing, Formal analysis. **Elisa Maio:** Writing – review & editing, Writing – original draft, Formal analysis. **Susannah Philip:** Formal analysis. **Jane Budd:** Writing – review & editing, Supervision. **Johannes Els:** Writing – review & editing, Writing – original draft.

## Declaration of competing interest

The authors declare that they have no known competing financial interests or personal relationships that could have appeared to influence the work reported in this paper.
